# Conserved 3′ UTR of Severe Acute Respiratory Syndrome Coronavirus 2: Potential Therapeutic Targets

**DOI:** 10.3389/fgene.2022.893141

**Published:** 2022-06-30

**Authors:** Jae Hyun Park, Jisook Moon

**Affiliations:** Department of Biotechnology, College of Life Science, CHA University, Seongnam, South Korea

**Keywords:** SARS-CoV-2, COVID-19, extracellular vesicles (EVs), virus variants, miRNA

## Abstract

Our previous paper showed that microRNAs (miRNAs) present within human placental or mesenchymal stem cell-derived extracellular vesicles (EVs) directly interacted with the RNA genome of severe acute respiratory syndrome coronavirus 2 (SARS-CoV-2), inhibiting viral replication. In this paper, we analyzed whether these miRNAs could exert antiviral activity against other variants of SARS-CoV-2. We downloaded compete SARS-CoV-2 genome data submitted to the National Center for Biotechnology Information for each SARS-CoV-2 variant, aligned the data to the reference SARS-CoV-2 genome sequence, and then confirmed the presence of 3′ untranslated region (UTR) mutations. We identified one type of 3′ UTR mutation in the Alpha variant, four in the Beta variant, four in the Gamma variant, three in the Delta variant, and none in the Omicron variant. Our findings indicate that 3′ UTR mutations rarely occur as persistent mutations. Interestingly, we further confirmed that this phenomenon could suppress virus replication in the same manner as the previously discovered interaction of placental-EV-derived miRNA with 3′ UTRs of SARS-CoV-2. Because the 3′ UTR of the SARS-CoV-2 RNA genome has almost no mutations, it is expected to be an effective therapeutic target regardless of future variants. Thus, a therapeutic strategy targeting the 3′ UTR of SARS-CoV-2 is likely to be extremely valuable, and such an approach is also expected to be applied to all RNA-based virus therapeutics.

## Introduction

As of April 2022, coronavirus 2019 (COVID-19) has infected >500 million people worldwide and has been reported as responsible for >6 million deaths. COVID-19 is caused by severe acute respiratory syndrome coronavirus 2 (SARS-CoV-2). This positive single-strand RNA virus has been identified as a variant of betacoronavirus. SARS-CoV-2 shows approximately 96% homology with bat coronavirus and 79.5% homology with SARS-CoV-1; it is the seventh human coronavirus discovered up to now (Zhou et al., 2020).

RNA viruses have a mutation rate 100–10,000 times higher than that of DNA viruses, and this high mutation rate is related to viral evolution as well as lethal mutagenesis (Duffy, 2018; Peck & Lauring, 2018). Thus, viruses with mutations related to viral replication, transformation, or immune system evasion have a competitive advantage over others, whereas mutations inappropriate for survival tend to be eliminated. In particular, the mutation of coronaviruses is slower than that of other RNA viruses, possibly because of a proofreading function. However, despite the low mutation rate of coronaviruses, novel variants have been reported (Callaway, 2020). A currently well-known SARS-CoV-2 mutation is the D614G spike protein mutation, which increases infectivity (Korber et al., 2020). According to William et al., whether mutations affecting the SARS-CoV-2 phenotype will break through infection- or vaccine-acquired immunity remains a point of contention; however, there is growing evidence that we should be prepared for mutations that can cause breakthrough infections (Harvey et al., 2021).

MicroRNAs (miRNAs) are small noncoding RNA molecules containing 18–25 bases that induce RNA degradation and translational suppression. miRNA can affect viral replication by direct interaction with the viral RNA genome or by acting on host mRNA (Trobaugh and Klimstra, 2017; Fani et al., 2018). For example, miRNAs can inhibit the replication of viruses, such as the human immunodeficiency virus 1, enterovirus 71, and hepatitis C virus (Jopling et al., 2005; Nathans et al., 2009; Zheng et al., 2013). In addition, approaches based on miRNA for SARS-CoV-2 have been recently reported (Alam and Lipovich, 2021; Fani et al., 2021; Hum et al., 2021).

Trobaugh et al. reported that eastern equine encephalitis virus replication was inhibited by host miR-142-3p, and the deletion of the miR-142-3p binding site on virus mutants was positively selected during virus replication. Furthermore, they suggested that there was an unknown mechanism at the corresponding binding site that was necessary for efficient virus replication (Trobaugh et al., 2014; Trobaugh and Klimstra, 2017).

Although the correlation between the conservation of miRNA binding sites in the viral 3′ untranslated region (UTR) and the life cycle of the virus is unclear, the secondary or tertiary structure of the 3′ UTR in RNA viruses is a necessary control element for RNA replication (Williams et al., 1999). In fact, Koyama et al., who analyzed 10,022 SARS-CoV-2 genomes that had been uploaded to databases by April 2020, reported two types of 3′ UTR mutations: 131 mutations of 29742G→T and 115 mutations of 29870C→A (Koyama et al., 2020). This extremely low mutation rate indicates that the SARS-CoV-2 3′ UTR region is much more stable (i.e., less susceptible to mutations) than other regions.

**FIGURE 1:**
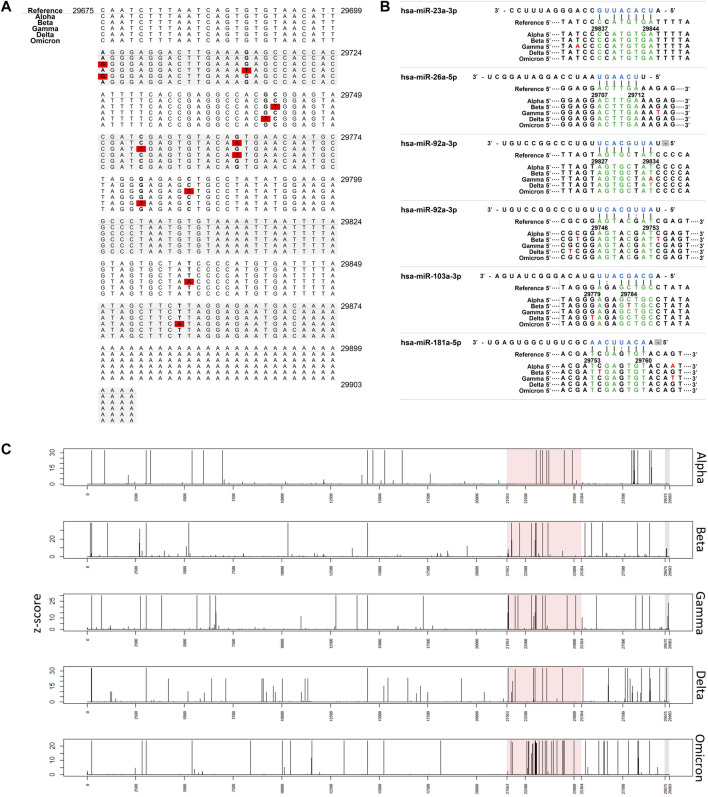
Interaction of miRNAs on SARS-CoV-2 3′ UTRs. **(A)** Nucleotide change in 3′ UTR of SARS-CoV-2 Red: nucleotide changes (associated with Table 2). **(B)** miRNA binding site in 3′ UTR of SARS-CoV-2. Nucleotide changes observed more than 1% were presented. **(C)** Plot showing the frequency of nucleotide changes in the SARS-CoV-2 genome. The frequency was scaled by the z-score within each variant. Red shading indicates spike glycoprotein coding region and grey shading indicates the 3′ UTR region.

In a previous study, we studied the antiviral effect of miRNAs based on the SARS-CoV-2 3′ UTR mutation stability and the potential interaction between miRNAs and the viral RNA genome. More specifically, we selected five miRNAs (miR-92a-3p, miR-26a-5p, miR-23a-3p, miR-103a-3p, and miR-181a-5p) present in placental extracellular vesicles (EVs) that were predicted to interact with the RNA genome of SARS-CoV-2. We also experimentally verified that the miRNAs bound to the complementary 3′ UTR of SARS-CoV-2 and inhibited viral replication (Figure 1A) (Park et al., 2021). Moreover, five miRNAs not only blocked SARS-CoV-2 replication but also regulated stress-induced inflammatory environment. Here, we further investigated whether the 3′ UTR of the SARS-CoV-2 binding site for EV miRNAs is conserved for various currently occurring variants using sequencing information submitted to the National Center for Biotechnology Information (NCBI) and predicted the potential effects of EV miRNAs on future variants.

## Materials and Methods

### Severe Acute Respiratory Syndrome Coronavirus 2 Complete Sequence Data

We analyzed sequence data selected by the World Health Organization for five variants of concern (Alpha, Beta, Delta, Gamma, and Omicron) as of December 2021. The SARS-CoV-2 reference genome (NC_045512.2) and complete genome sequence data of SARS-CoV-2 were downloaded from the NCBI and NCBI Virus databases, respectively. The sequence data were selected using the following filters: txid2697049; nucleotide completeness: complete; and collection dates: ∼2021-12-17.

Each variant was sorted according to the Pango lineage classification option of NCBI Virus. The Pango lineages used for the search were B.1.1.7, B.1.351, P.1, B.1.617.2, and BA.1 for the Alpha, Beta, Delta, Gama, and Omicron variants, respectively (Table 1).

**TABLE 1:** SARS-CoV-2 sample information.

Label	Pango lineage	Date	Sample	QC passed*
Alpha	B.1.1.7	∼2021-12-15	194,972	174,209
Beta	B.1.351	∼2021-12-17	563	479
Gamma	P.1	∼2021-12-17	6,277	6,229
Delta	B.1.617.2	∼2021-12-21	12,162	11,432
Omicron	BA.1	∼2021-12-20	195	191

* Sequences with >1% of ambiguous bases were removed.

### Sequencing Data Alignment and Analysis

We performed multiple sequence alignment against the SARS-CoV-2 reference genome using MAFFT software version 7.487 (Nakamura et al., 2018), which can rapidly calculate full-length multiple sequence alignments of closely-related viral genomes. Sequences with >1% of ambiguous bases were removed. For 3′ UTR region analysis, we extracted the sequences aligned between 29,675 and 29,903 in the SARS-CoV-2 reference genome (range of the 3′ UTR).

### Prediction of MiRNA-Viral 3′ Untranslated Region Interaction

We used the PITA tool (Kertesz et al., 2007) to investigate and predict the miRNA binding sites in the 3′ UTR of SARS-CoV-2, using the SARS-CoV-2 complete genome sequence (NC_045512.2), from which the 3′ UTR sequence was also extracted.

### Graphics

Plots were drawn using R programming language. The frequency of nucleotide changes in the SARS-CoV-2 genome was scaled by the z-score.

## Results

We analyzed the following sequence data obtained from the NCBI database: 194,972 Alpha variants of SARS-CoV-2 (Pango lineage B.1.1.7), 563 Beta variants (B.1.351), 6277 Gamma variants (P.1), 12,162 Delta variants (B.1.617.2), and 195 Omicron variants (BA.1) (Table 1). Sequences with >1% of ambiguous bases were removed. We aligned the sequencing data obtained from NCBI to the SARS-CoV-2 reference genome (accession number: NC_045512.2) using the MAFFT Tool (Nakamura et al., 2018), and through this, the position where the mutation occurred for each variant was determined. We first investigated nucleotide changes at the 3′ UTR of SARS-CoV-2. Although we found 348 cases of nucleotide changes in the Alpha variant, 17 in the Beta variant, 86 in the Gamma variant, 138 in the Delta variant, and 3 in the Omicron variant. The frequency of nucleotide changes in the 3′ UTR was very low in five variants. We further investigated the nucleotide changes with a frequency of >1% in all samples for each variant, and the following nucleotide changes were identified: 29764G→A in the Alpha variant; 29754C→T, 29743C→T, 29700A→G, and 29784C→T in the Beta variant; 29834T→A, 29858T→A, 29764G→T, and 29715G→T in the Gamma variant; and 29742G→T, 29779G→T, and 29700A→G in the Delta variant (Figure 1A and Table 2). Table 2 shows nucleotide changes that were observed in more than 1%. We identified that nucleotide changes in the 3′ UTR had a lower frequency of mutation compared to the spike glycoprotein coding region currently undergoing rapid mutation. In the Omicron variant, one case each of 29742G→T, 29772T→C, and 29818A→T mutations located in the 3′ UTR of SARS-CoV-2 were found, but in all three cases, the frequency was <1% of the total samples (n = 191). It must be taken into account that in the case of the Omicron variant, the sample size was small as of December 2021, so continuous monitoring and analysis are required. All nucleotide changes that we identified are presented in Supplementary Table S1.

**TABLE 2:** Number of nucleotide changes in the 3′ UTR of SARS-CoV-2.

Variant	Tested sample	Spike glycoprotein coding region nucleotide change		3′ UTR nucleotide change	
		Change	Number of samples	Change	Number of samples
Alpha	174,209	23403A→G	174185	29764G→A	4,161
		24506T→G	174168		
		23709C→T	174160		
		23271C→A	174152		
		24914G→C	174141		
		23604C→A	174116		
		23063A→T	173889		
		25135G→T	18281		
		21855C→T	3531		
		21575C→T	3410		
		21614C→T	2342		
		21974G→C	2160		
Beta	479	21801A→C	479	29754C→T	114
		23403A→G	479	29743C→T	113
		23664C→T	479	29700A→G	7
		22206A→G	478	29784C→T	7
		23063A→T	476		
		23012G→A	475		
		22813G→T	472		
		21614C→T	107		
		24415G→A	80		
		23198T→C	45		
		21641G→T	29		
		23764A→T	29		
		25088G→T	21		
		21574T→C	9		
		21618C→T	7		
		21636C→T	7		
		22119T→C	6		
		23029C→T	6		
		21974G→T	5		
		23248C→T	5		
		23470T→C	5		
		25378C→T	5		
Gamma	6,229	23403A→G	6226	29834T→A	4,849
		23063A→T	6221	29858T→A	248
		22812A→C	6220	29764G→T	135
		23012G→A	6217	29715G→T	63
		23525C→T	6214		
		21638C→T	6210		
		21614C→T	6202		
		22132G→T	6181		
		25088G→T	6181		
		24642C→T	6170		
		21621C→A	6146		
		21974G→T	6124		
		22945C→T	2409		
		23625C→T	706		
		22841G→A	243		
		22456A→G	224		
		23611G→T	211		
		25159T→C	160		
		21597C→T	135		
		23005T→C	133		
		22211C→T	108		
		21724G→T	69		
Delta	11,432	23403A→G	11423	29742G→T	11,346
		21618C→G	11416	29779G→T	119
		22917T→G	11413	29700A→G	121
		22995C→A	11412		
		23604C→G	11409		
		24410G→A	11408		
		21987G→A	10993		
		21846C→T	7025		
		21792A→C	3302		
		24130C→T	1088		
		22936G→A	887		
		21575C→T	211		
		21595C→T	172		
		23741C→T	139		
		21806C→A	130		
		22227C→T	129		
		21811C→T	124		
		25062G→T	115		
Omicron	191	22992G→A	191		
		22995C→A	191		
		23202C→A	191		
		23403A→G	191		
		23525C→T	191		
		23599T→G	191		
		23604C→A	191		
		24424A→T	191		
		24469T→A	191		
		24503C→T	191		
		21762C→T	190		
		23013A→C	190		
		24130C→A	190		
		25000C→T	190		
		23948G→T	189		
		22578G→A	188		
		22679T→C	187		
		22686C→T	187		
		23854C→A	186		
		22673T→C	185		
		22674C→T	185		
		23040A→G	182		
		21846C→T	181		
		23048G→A	180		
		23063A→T	177		
		23055A→G	176		
		23075T→C	176		
		22898G→A	174		
		22882T→G	172		
		22813G→T	120		
		22599G→A	37		
		21595C→T	18		
		23664C→T	16		
		21766A→C	3		
		22917T→G	3		
		21995T→G	2		
		22006C→A	2		
		22120C→T	2		

We used a miRNA binding prediction tool to further investigate how these 3′ UTR mutations affect miRNA binding (Table 3). Table 3 shows thermodynamic energy required for binding (kcal/mol). Lower energy indicates stronger binding prediction. In a recent study from our laboratory (Park et al., 2021), five miRNAs (hsa-miR-103a-3p, hsa-miR-181a-5p, hsa-miR-23a-5p, hsa-miR-26a-5p, and hsa-miR-92a-3p) were predicted and experimentally verified to interact with mutant 3′ UTR sites of SARS-CoV-2 reference sequences (Figure 1B). Most of the interactions between these miRNAs and the mutation sites on 3′ UTRs of the SARS-CoV-2 genome did not change their binding energy when compared with interactions between the reference 3′ UTRs of the SARS-CoV-2 genome. However, the binding of hsa-miR-103a-3p in the Beta variant and hsa-miR-92a-3p in the Gamma variant to the 29784C→T and 29834T→A mutations, respectively, of the 3′ UTR of SARS-CoV-2 genome was not predicted. Notably, the binding affinity to hsa-miR-181a-5p was increased compared with the reference sequence in the 29754C→T mutation in the Beta variant. These findings suggest that the miRNA binding site can disappear as the result of specific 3′ UTR mutations of SARS-CoV-2, consequently diminishing most of the related miRNA affinity. However, in certain miRNAs, the binding affinity is predicted to increase despite the site change due to mutation, suggesting that efficacy may vary depending on miRNA characteristics (Table 3).

**TABLE 3:** Binding energy prediction in 3′ UTRs of SARS-CoV-2.

Variant	Nucleotide change	Hsa-miR-103a-3p	Hsa-miR-181a-5p	Hsa-miR-23a-5p	Hsa-miR-26a-5p	Hsa-miR-92a-3p (site 1)	Hsa-miR-92a-3p (site 2)
Reference	—	−12.6	−18.7	−15.7	−14.9	−13.8	−9.01
Alpha	29764G→A						
Beta	29754C→T		−21.1				
	29743C→T		−16.91			−13.5	
	29700A→G				−16.1		
	29784C→T	Not predicted					
Delta	29700A→G				−16.1		
	29742G→T		−16.91			−13.5	
	29779G→T	−13.1					
Gamma	29715G→T						
	29764G→T						
	29834T→A						Not predicted
	29858T→A						
All*		Not predicted	−19.46	−15.7	−16.1	−13.5	Not predicted

* The predicted binding energy when all nucleotide changes in Table 2 occur at the same time.

Subsequently, to determine whether the miRNA binding site in the 3′ UTR is conserved among variants, we investigated the frequency of mutations in the human miRNA binding site in the SARS-CoV-2 3′ UTR (Figure 1B). The colored spot in Figure 1B shows the site where many nucleotide changes have been detected. In the Alpha variant, mutations were found within the miRNA binding site, but the frequency was extremely low (<1%). Furthermore, hardly any mutations in the hsa-miR-23a-3p binding site were found in the five variants. This suggests that hsa-miR-23a-3p can be used as a therapeutic agent regardless of the SARS-CoV-2 variant.

Subsequently, to measure how often nucleotide changes occur in the 3′ UTR, we investigated the frequency of nucleotide changes in the whole SARS-CoV-2 genome to determine the relative frequency of 3′ UTR nucleotide changes (Figure 1C). Mutations in 3′ UTR were relatively minor when compared with the frequency of nucleotide changes in the whole genome. In particular, nucleotide changes in the 3′ UTR region were detected less frequently than those in the spike glycoprotein coding region. Major mutations in the 3′ UTR were only found in the Beta, Gamma, and Delta variants.

## Discussion

We found a major UTR mutation in the 3′ UTR in the SARS-CoV-2 Gamma and Delta variants. In the Gamma variant, the 29834T→A mutation accounted for 78% of the analyzed cases, and in the Delta variant, the 29742G→T mutation accounted for 99% of the analyzed cases. The effect of these 3′ UTR mutations on the replication and infectivity of the Gamma and Delta variants is unknown. We speculated that they would not affect the binding affinity of the five miRNAs (miR-92a-3p, miR-26a-5p, miR-23a-3p, miR-103a-3p, and miR-181a-5p) because we previously identified antiviral effects when these five miRNAs bound to the SARS-CoV-2 3′ UTR region (Figure 1B).

Meanwhile, the 29784C→T and 29834T→A mutations in the SARS-CoV-2 3′ UTR region of the Beta and Gamma variants were predicted to inhibit the binding affinity of hsa-miR-103a-3p and hsa-miR-92a-3p, respectively. These are sites of attachment for the seed regions of miRNAs, which play an essential role in miRNA-RNA interaction. Analysis of the match of these seed regions using the miRNA binding prediction tool revealed 3′ UTR mismatches. Although hsa-miR-92a-3p was not predicted to bind at position 29834 of the SARS-CoV-2 3′ UTR, it was predicted to bind to another site in the SARS-CoV-2 3′ UTR (Table 3), and this site was unaffected by the 29834T→A 3′ UTR mutation. Additionally, because the other binding site has a higher binding affinity with hsa-miR-92a-3p than the site at position 29834, it plays a more prominent role in the interaction between hsa-miR-92a-3p and the SARS-CoV-2 3′ UTR than the binding site at position 29834. In a previous study, we observed that a combination treatment of miRNAs was more effective against the 3′ UTR of SARS-CoV-2, suggesting that there is a synergistic effect on the miRNAs. We suspect that the synergistic effect on the miRNAs, which may be attributed to different binding sites.

Although the antiviral efficacy of miRNAs against SARS-CoV-2 has been predicted despite mutations that have occurred thus far, there are some miRNAs whose binding affinity is predicted to change due to mutation. Regarding the binding of hsa-miR-181-a-5p, the 29743C→T mutation of the Beta variant and the 29742G→T mutation of the Delta variant are expected to slightly decrease the binding affinity. In contrast, the binding energy of hsa-miR-181-a-5p is predicted to be stronger in the 29754C→T mutation of the Beta variant. Thus, changes in the binding affinity of miRNAs according to variants will be a major consideration when establishing an antiviral treatment strategy using miRNAs for COVID-19 in the future.

It was revealed that the binding sites of the five miRNAs that we previously reported as showing an antiviral effect (Park et al., 2021) were mostly conserved in other variants, even in newly occurring SARS-CoV-2 variants. These findings predict that EV- or miRNA-based antiviral therapy will be effective against other major variants of SARS-CoV-2 that are likely to continue to occur in the future.

The current study did not investigate whether the reason why these five miRNA binding sites are mostly conserved across variants is to preserve the 3′ UTR function or whether there is an unknown mechanism, as Trobaugh et al. suggested (Trobaugh et al., 2014; Trobaugh and Klimstra, 2017). Although it is necessary to experimentally verify the antiviral efficacy of a therapeutic strategy targeting the 3′ UTR of SARS-CoV-2, our evidence convincingly shows the potential of miRNAs as the most appropriate antiviral treatment to counter the emergence of virus variants, which is the most significant problem.

## Data Availability

Publicly available datasets were analyzed in this study. This data can be found here: https://www.ncbi.nlm.nih.gov/labs/virus/
