# Supplementary material for: Conserved 3′ UTR of Severe Acute Respiratory Syndrome Coronavirus 2: Potential Therapeutic Targets
Source: Front Genet. 2022 Jun 30;13:893141. doi: 10.3389/fgene.2022.893141 (PMC9280349; doi:10.3389/fgene.2022.893141)
Supplement: Supplementary file 1 [file Table1.DOCX]

**Supplementary Table 1. List of all detected nucleotide changes in SARS-CoV-2 3′ UTRs**

| Mutation | Alpha | Beta | Gamma | Delta | Omicron |
| --- | --- | --- | --- | --- | --- |
| 29675C→T | 83 | 0 | 7 | 4 | 0 |
| 29676A→G | 2 | 0 | 0 | 0 | 0 |
| 29676A→T | 21 | 0 | 0 | 0 | 0 |
| 29677A→C | 2 | 0 | 0 | 0 | 0 |
| 29677A→G | 2 | 0 | 0 | 1 | 0 |
| 29678T→A | 1 | 0 | 0 | 0 | 0 |
| 29678T→C | 5 | 0 | 0 | 1 | 0 |
| 29679C→T | 105 | 0 | 4 | 2 | 0 |
| 29680T→A | 1 | 0 | 0 | 0 | 0 |
| 29680T→C | 1 | 0 | 0 | 0 | 0 |
| 29680T→G | 0 | 0 | 0 | 4 | 0 |
| 29681T→A | 1 | 0 | 0 | 0 | 0 |
| 29681T→C | 4 | 0 | 0 | 0 | 0 |
| 29682T→A | 1 | 0 | 0 | 0 | 0 |
| 29682T→C | 7 | 0 | 0 | 0 | 0 |
| 29683A→C | 1 | 0 | 0 | 0 | 0 |
| 29683A→G | 14 | 0 | 0 | 0 | 0 |
| 29684A→G | 6 | 0 | 0 | 0 | 0 |
| 29685T→A | 6 | 0 | 0 | 0 | 0 |
| 29685T→C | 95 | 0 | 0 | 0 | 0 |
| 29685T→G | 5 | 0 | 0 | 0 | 0 |
| 29686C→G | 172 | 0 | 0 | 0 | 0 |
| 29686C→T | 247 | 1 | 1 | 11 | 0 |
| 29688G→A | 2 | 0 | 0 | 0 | 0 |
| 29688G→C | 8 | 0 | 0 | 0 | 0 |
| 29688G→T | 117 | 0 | 2 | 17 | 0 |
| 29689T→C | 2 | 0 | 0 | 0 | 0 |
| 29690G→A | 1 | 0 | 0 | 0 | 0 |
| 29690G→C | 1 | 0 | 0 | 0 | 0 |
| 29690G→T | 170 | 0 | 3 | 7 | 0 |
| 29691T→G | 4 | 0 | 0 | 0 | 0 |
| 29692G→A | 1 | 0 | 1 | 0 | 0 |
| 29692G→C | 13 | 0 | 0 | 0 | 0 |
| 29692G→T | 83 | 0 | 0 | 1 | 0 |
| 29693T→C | 12 | 0 | 0 | 0 | 0 |
| 29694A→G | 2 | 0 | 0 | 1 | 0 |
| 29695A→G | 5 | 0 | 0 | 0 | 0 |
| 29696C→A | 2 | 0 | 0 | 0 | 0 |
| 29696C→T | 19 | 0 | 0 | 0 | 0 |
| 29697A→G | 2 | 0 | 0 | 0 | 0 |
| 29698T→C | 2 | 0 | 0 | 0 | 0 |
| 29698T→G | 3 | 0 | 0 | 0 | 0 |
| 29700A→G | 250 | 7 | 4 | 121 | 0 |
| 29701G→A | 4 | 0 | 0 | 0 | 0 |
| 29701G→T | 11 | 0 | 1 | 1 | 0 |
| 29702G→A | 22 | 0 | 0 | 5 | 0 |
| 29702G→T | 3 | 0 | 0 | 0 | 0 |
| 29703G→A | 1 | 0 | 0 | 1 | 0 |
| 29703G→T | 9 | 0 | 1 | 0 | 0 |
| 29704A→C | 1 | 0 | 0 | 0 | 0 |
| 29704A→G | 0 | 0 | 0 | 4 | 0 |
| 29705G→C | 1 | 0 | 0 | 0 | 0 |
| 29705G→T | 17 | 0 | 0 | 26 | 0 |
| 29706G→A | 34 | 0 | 2 | 1 | 0 |
| 29706G→C | 14 | 0 | 0 | 0 | 0 |
| 29706G→T | 242 | 2 | 2 | 13 | 0 |
| 29707A→G | 2 | 0 | 0 | 11 | 0 |
| 29708C→A | 2 | 0 | 0 | 0 | 0 |
| 29708C→G | 1 | 0 | 0 | 0 | 0 |
| 29708C→T | 207 | 0 | 2 | 9 | 0 |
| 29710T→A | 3 | 0 | 0 | 0 | 0 |
| 29710T→C | 3 | 0 | 0 | 23 | 0 |
| 29711G→A | 7 | 0 | 0 | 0 | 0 |
| 29711G→C | 9 | 0 | 0 | 0 | 0 |
| 29711G→T | 235 | 0 | 4 | 5 | 0 |
| 29712A→C | 0 | 0 | 0 | 30 | 0 |
| 29712A→G | 1 | 0 | 0 | 0 | 0 |
| 29713A→G | 1 | 0 | 0 | 0 | 0 |
| 29714A→G | 1 | 0 | 0 | 0 | 0 |
| 29714A→T | 1 | 0 | 0 | 0 | 0 |
| 29715G→A | 2 | 0 | 0 | 4 | 0 |
| 29715G→T | 82 | 0 | 63 | 3 | 0 |
| 29716A→G | 3 | 0 | 0 | 0 | 0 |
| 29716A→T | 2 | 0 | 0 | 0 | 0 |
| 29717G→A | 88 | 0 | 0 | 1 | 0 |
| 29717G→C | 2 | 0 | 0 | 0 | 0 |
| 29717G→T | 28 | 0 | 0 | 0 | 0 |
| 29718C→T | 13 | 1 | 0 | 1 | 0 |
| 29719C→A | 7 | 0 | 0 | 3 | 0 |
| 29719C→T | 35 | 0 | 0 | 0 | 0 |
| 29720A→G | 2 | 0 | 0 | 1 | 0 |
| 29720A→T | 3 | 0 | 0 | 0 | 0 |
| 29721C→A | 6 | 0 | 0 | 0 | 0 |
| 29721C→T | 100 | 1 | 0 | 11 | 0 |
| 29722C→A | 2 | 0 | 0 | 0 | 0 |
| 29722C→T | 966 | 0 | 1 | 2 | 0 |
| 29723A→G | 3 | 0 | 0 | 0 | 0 |
| 29723A→T | 3 | 0 | 0 | 0 | 0 |
| 29724C→T | 144 | 0 | 1 | 3 | 0 |
| 29725A→G | 3 | 0 | 0 | 0 | 0 |
| 29725A→T | 1 | 0 | 0 | 3 | 0 |
| 29726T→A | 2 | 0 | 0 | 0 | 0 |
| 29726T→C | 9 | 0 | 0 | 1 | 0 |
| 29727T→C | 4 | 0 | 0 | 0 | 0 |
| 29727T→G | 1 | 0 | 0 | 0 | 0 |
| 29728T→A | 177 | 0 | 0 | 0 | 0 |
| 29728T→C | 1 | 0 | 0 | 0 | 0 |
| 29728T→G | 3 | 0 | 0 | 0 | 0 |
| 29729T→A | 6 | 0 | 0 | 0 | 0 |
| 29729T→C | 1 | 0 | 0 | 5 | 0 |
| 29730C→G | 5 | 0 | 0 | 0 | 0 |
| 29730C→T | 94 | 0 | 3 | 4 | 0 |
| 29731A→G | 39 | 0 | 1 | 0 | 0 |
| 29731A→T | 4 | 0 | 0 | 0 | 0 |
| 29732C→A | 1 | 0 | 0 | 0 | 0 |
| 29732C→T | 39 | 0 | 2 | 0 | 0 |
| 29733C→A | 10 | 0 | 0 | 3 | 0 |
| 29733C→T | 298 | 2 | 4 | 30 | 0 |
| 29734G→A | 10 | 0 | 0 | 2 | 0 |
| 29734G→C | 25 | 0 | 0 | 4 | 0 |
| 29734G→T | 109 | 2 | 1 | 4 | 0 |
| 29735A→C | 1 | 0 | 0 | 0 | 0 |
| 29735A→G | 114 | 0 | 0 | 0 | 0 |
| 29736G→A | 30 | 0 | 0 | 1 | 0 |
| 29736G→T | 108 | 0 | 2 | 20 | 0 |
| 29737G→A | 126 | 0 | 2 | 2 | 0 |
| 29737G→C | 2 | 0 | 0 | 0 | 0 |
| 29737G→T | 105 | 0 | 6 | 14 | 0 |
| 29738C→A | 22 | 0 | 0 | 5 | 0 |
| 29738C→T | 142 | 2 | 5 | 26 | 0 |
| 29739C→T | 170 | 0 | 0 | 4 | 0 |
| 29740A→G | 68 | 0 | 0 | 3 | 0 |
| 29740A→T | 2 | 0 | 0 | 0 | 0 |
| 29741C→A | 7 | 0 | 0 | 65 | 0 |
| 29741C→T | 203 | 1 | 11 | 5 | 0 |
| 29742G→A | 130 | 0 | 24 | 0 | 0 |
| 29742G→C | 2 | 0 | 0 | 0 | 0 |
| 29742G→T | 216 | 0 | 5 | 11346 | 1 |
| 29743C→A | 1 | 0 | 0 | 0 | 0 |
| 29743C→T | 343 | 113 | 33 | 12 | 0 |
| 29744G→A | 101 | 0 | 0 | 24 | 0 |
| 29744G→T | 145 | 0 | 2 | 12 | 0 |
| 29745G→A | 3 | 0 | 0 | 1 | 0 |
| 29745G→C | 1 | 0 | 0 | 0 | 0 |
| 29745G→T | 92 | 0 | 5 | 1 | 0 |
| 29746A→C | 2 | 0 | 0 | 0 | 0 |
| 29746A→G | 1 | 0 | 0 | 4 | 0 |
| 29746A→T | 3 | 0 | 0 | 0 | 0 |
| 29747G→A | 3 | 0 | 0 | 1 | 0 |
| 29747G→C | 12 | 0 | 0 | 0 | 0 |
| 29747G→T | 126 | 0 | 6 | 64 | 0 |
| 29748T→C | 1 | 0 | 0 | 1 | 0 |
| 29749A→G | 10 | 0 | 0 | 0 | 0 |
| 29749A→T | 2 | 0 | 0 | 0 | 0 |
| 29750C→A | 1 | 0 | 0 | 3 | 0 |
| 29750C→T | 356 | 0 | 49 | 51 | 0 |
| 29751G→A | 36 | 0 | 0 | 2 | 0 |
| 29751G→C | 129 | 0 | 0 | 8 | 0 |
| 29751G→T | 77 | 0 | 2 | 5 | 0 |
| 29752A→G | 7 | 0 | 0 | 5 | 0 |
| 29752A→T | 36 | 0 | 0 | 0 | 0 |
| 29753T→C | 21 | 0 | 1 | 1 | 0 |
| 29753T→G | 1 | 0 | 0 | 2 | 0 |
| 29754C→A | 5 | 0 | 0 | 0 | 0 |
| 29754C→T | 121 | 114 | 0 | 9 | 0 |
| 29755G→C | 4 | 0 | 0 | 0 | 0 |
| 29755G→T | 63 | 0 | 3 | 0 | 0 |
| 29756A→C | 2 | 0 | 0 | 0 | 0 |
| 29756A→G | 4 | 0 | 0 | 12 | 0 |
| 29756A→T | 3 | 0 | 0 | 7 | 0 |
| 29757G→A | 0 | 0 | 0 | 1 | 0 |
| 29757G→T | 91 | 0 | 2 | 1 | 0 |
| 29758T→C | 1 | 0 | 1 | 0 | 0 |
| 29758T→G | 2 | 0 | 1 | 0 | 0 |
| 29759G→A | 3 | 0 | 1 | 0 | 0 |
| 29759G→C | 9 | 0 | 0 | 0 | 0 |
| 29759G→T | 267 | 0 | 2 | 5 | 0 |
| 29760T→A | 2 | 0 | 0 | 0 | 0 |
| 29760T→C | 82 | 0 | 0 | 0 | 0 |
| 29760T→G | 10 | 0 | 0 | 0 | 0 |
| 29761A→G | 2 | 0 | 0 | 0 | 0 |
| 29761A→T | 1 | 0 | 0 | 0 | 0 |
| 29762C→G | 1 | 0 | 0 | 0 | 0 |
| 29762C→T | 87 | 1 | 13 | 17 | 0 |
| 29763A→C | 2 | 0 | 0 | 0 | 0 |
| 29763A→G | 5 | 0 | 0 | 0 | 0 |
| 29763A→T | 19 | 0 | 0 | 0 | 0 |
| 29764G→A | 4161 | 0 | 0 | 1 | 0 |
| 29764G→C | 22 | 0 | 0 | 0 | 0 |
| 29764G→T | 78 | 0 | 135 | 8 | 0 |
| 29765T→A | 1 | 0 | 0 | 0 | 0 |
| 29765T→C | 62 | 0 | 0 | 1 | 0 |
| 29766G→A | 7 | 0 | 2 | 0 | 0 |
| 29766G→C | 23 | 0 | 0 | 0 | 0 |
| 29766G→T | 17 | 0 | 0 | 4 | 0 |
| 29767A→C | 2 | 0 | 1 | 0 | 0 |
| 29767A→G | 34 | 0 | 0 | 0 | 0 |
| 29768A→G | 8 | 0 | 0 | 0 | 0 |
| 29768A→T | 2 | 0 | 1 | 0 | 0 |
| 29769C→A | 3 | 0 | 0 | 0 | 0 |
| 29769C→G | 2 | 0 | 0 | 0 | 0 |
| 29769C→T | 202 | 0 | 4 | 34 | 0 |
| 29770A→G | 9 | 0 | 0 | 0 | 0 |
| 29770A→T | 0 | 0 | 3 | 0 | 0 |
| 29771A→G | 22 | 0 | 0 | 21 | 0 |
| 29771A→T | 0 | 0 | 1 | 0 | 0 |
| 29772T→A | 3 | 0 | 0 | 0 | 0 |
| 29772T→C | 3 | 0 | 0 | 7 | 1 |
| 29772T→G | 1 | 0 | 0 | 0 | 0 |
| 29773G→A | 1 | 0 | 1 | 0 | 0 |
| 29773G→T | 53 | 0 | 1 | 2 | 0 |
| 29774C→A | 8 | 0 | 0 | 2 | 0 |
| 29774C→G | 2 | 0 | 0 | 0 | 0 |
| 29774C→T | 547 | 0 | 3 | 75 | 0 |
| 29775T→C | 7 | 0 | 0 | 0 | 0 |
| 29776A→G | 1 | 0 | 0 | 0 | 0 |
| 29777G→A | 3 | 0 | 0 | 8 | 0 |
| 29777G→C | 1 | 0 | 0 | 0 | 0 |
| 29777G→T | 105 | 1 | 0 | 11 | 0 |
| 29778G→A | 15 | 0 | 0 | 1 | 0 |
| 29778G→T | 17 | 0 | 2 | 1 | 0 |
| 29779G→A | 5 | 0 | 0 | 0 | 0 |
| 29779G→C | 1 | 0 | 0 | 0 | 0 |
| 29779G→T | 213 | 1 | 0 | 119 | 0 |
| 29780A→C | 2 | 0 | 0 | 0 | 0 |
| 29780A→G | 6 | 0 | 0 | 0 | 0 |
| 29780A→T | 8 | 0 | 0 | 0 | 0 |
| 29781G→A | 4 | 0 | 2 | 1 | 0 |
| 29781G→C | 2 | 0 | 0 | 1 | 0 |
| 29781G→T | 208 | 0 | 2 | 13 | 0 |
| 29782A→G | 9 | 0 | 0 | 4 | 0 |
| 29783G→A | 1 | 0 | 0 | 0 | 0 |
| 29783G→T | 2 | 0 | 0 | 0 | 0 |
| 29784C→A | 2 | 0 | 0 | 0 | 0 |
| 29784C→T | 219 | 7 | 4 | 7 | 0 |
| 29785T→A | 2 | 0 | 0 | 0 | 0 |
| 29785T→C | 25 | 0 | 0 | 0 | 0 |
| 29785T→G | 2 | 0 | 0 | 0 | 0 |
| 29787C→T | 1 | 0 | 0 | 0 | 0 |
| 29788C→T | 0 | 0 | 0 | 1 | 0 |
| 29789T→A | 17 | 0 | 0 | 0 | 0 |
| 29789T→C | 6 | 0 | 0 | 0 | 0 |
| 29790A→G | 0 | 0 | 0 | 4 | 0 |
| 29791T→C | 6 | 0 | 0 | 0 | 0 |
| 29792A→G | 1 | 0 | 0 | 0 | 0 |
| 29792A→T | 1 | 0 | 0 | 0 | 0 |
| 29793T→A | 6 | 0 | 2 | 0 | 0 |
| 29793T→C | 4 | 0 | 0 | 0 | 0 |
| 29793T→G | 1 | 0 | 0 | 0 | 0 |
| 29795G→A | 1 | 0 | 2 | 0 | 0 |
| 29797A→G | 9 | 0 | 0 | 1 | 0 |
| 29797A→T | 1 | 0 | 0 | 0 | 0 |
| 29798G→T | 1 | 0 | 0 | 0 | 0 |
| 29799A→C | 1 | 0 | 0 | 0 | 0 |
| 29799A→G | 6 | 0 | 0 | 0 | 0 |
| 29800G→A | 8 | 0 | 0 | 1 | 0 |
| 29800G→T | 22 | 0 | 0 | 0 | 0 |
| 29801C→A | 1 | 0 | 0 | 1 | 0 |
| 29801C→T | 7 | 0 | 60 | 0 | 0 |
| 29802C→T | 95 | 0 | 1 | 1 | 0 |
| 29803C→T | 76 | 0 | 0 | 8 | 0 |
| 29804T→C | 37 | 0 | 0 | 0 | 0 |
| 29804T→G | 1 | 0 | 0 | 3 | 0 |
| 29805A→G | 0 | 0 | 0 | 7 | 0 |
| 29805A→T | 2 | 0 | 1 | 3 | 0 |
| 29806A→G | 19 | 0 | 0 | 22 | 0 |
| 29807T→A | 4 | 0 | 0 | 1 | 0 |
| 29807T→C | 3 | 0 | 0 | 0 | 0 |
| 29807T→G | 1 | 0 | 0 | 0 | 0 |
| 29808G→A | 2 | 0 | 0 | 0 | 0 |
| 29808G→C | 1 | 0 | 0 | 0 | 0 |
| 29808G→T | 39 | 0 | 0 | 0 | 0 |
| 29810G→A | 5 | 0 | 0 | 0 | 0 |
| 29810G→T | 127 | 1 | 3 | 2 | 0 |
| 29811T→C | 6 | 0 | 0 | 0 | 0 |
| 29813A→G | 0 | 0 | 0 | 2 | 0 |
| 29815A→C | 2 | 0 | 0 | 0 | 0 |
| 29815A→G | 1 | 0 | 0 | 0 | 0 |
| 29816T→C | 1 | 0 | 0 | 0 | 0 |
| 29817T→C | 2 | 0 | 0 | 0 | 0 |
| 29818A→G | 14 | 0 | 0 | 0 | 0 |
| 29818A→T | 0 | 0 | 0 | 0 | 1 |
| 29819A→C | 2 | 0 | 0 | 0 | 0 |
| 29820T→C | 1 | 0 | 0 | 0 | 0 |
| 29820T→G | 6 | 0 | 0 | 0 | 0 |
| 29821T→C | 3 | 0 | 0 | 0 | 0 |
| 29821T→G | 1 | 1 | 0 | 0 | 0 |
| 29822T→C | 24 | 0 | 0 | 0 | 0 |
| 29822T→G | 1 | 0 | 0 | 0 | 0 |
| 29824A→G | 1 | 0 | 0 | 0 | 0 |
| 29824A→T | 7 | 0 | 0 | 0 | 0 |
| 29825G→A | 50 | 0 | 0 | 1 | 0 |
| 29825G→C | 4 | 0 | 0 | 0 | 0 |
| 29825G→T | 92 | 0 | 2 | 1 | 0 |
| 29826T→C | 2 | 0 | 0 | 0 | 0 |
| 29826T→G | 4 | 0 | 0 | 0 | 0 |
| 29827A→T | 3 | 0 | 0 | 0 | 0 |
| 29828G→A | 3 | 0 | 1 | 0 | 0 |
| 29828G→T | 14 | 0 | 11 | 0 | 0 |
| 29829T→A | 3 | 0 | 0 | 0 | 0 |
| 29830G→A | 1 | 0 | 0 | 0 | 0 |
| 29830G→C | 0 | 0 | 1 | 0 | 0 |
| 29830G→T | 8 | 0 | 0 | 0 | 0 |
| 29831C→A | 1 | 0 | 0 | 0 | 0 |
| 29831C→T | 13 | 0 | 0 | 2 | 0 |
| 29832T→C | 4 | 0 | 1 | 1 | 0 |
| 29833A→C | 0 | 0 | 0 | 1 | 0 |
| 29833A→G | 4 | 0 | 1 | 0 | 0 |
| 29834T→A | 2 | 0 | 4849 | 0 | 0 |
| 29834T→C | 18 | 0 | 1 | 0 | 0 |
| 29834T→G | 3 | 0 | 0 | 0 | 0 |
| 29835C→A | 5 | 0 | 0 | 0 | 0 |
| 29835C→T | 108 | 0 | 1 | 8 | 0 |
| 29836C→A | 0 | 0 | 31 | 0 | 0 |
| 29836C→T | 27 | 0 | 0 | 2 | 0 |
| 29837C→A | 4 | 0 | 1 | 0 | 0 |
| 29837C→G | 1 | 0 | 0 | 0 | 0 |
| 29837C→T | 41 | 0 | 1 | 8 | 0 |
| 29838C→T | 14 | 0 | 1 | 11 | 0 |
| 29839A→C | 0 | 0 | 0 | 1 | 0 |
| 29839A→T | 1 | 0 | 0 | 0 | 0 |
| 29840T→C | 12 | 0 | 0 | 0 | 0 |
| 29841G→T | 0 | 0 | 0 | 1 | 0 |
| 29842T→A | 0 | 0 | 0 | 1 | 0 |
| 29843G→T | 6 | 0 | 0 | 0 | 0 |
| 29846T→C | 1 | 0 | 0 | 0 | 0 |
| 29847T→C | 1 | 0 | 0 | 0 | 0 |
| 29848T→A | 3 | 0 | 0 | 0 | 0 |
| 29850A→C | 1 | 0 | 0 | 0 | 0 |
| 29850A→G | 1 | 0 | 0 | 0 | 0 |
| 29850A→T | 2 | 0 | 0 | 0 | 0 |
| 29851T→C | 4 | 0 | 0 | 0 | 0 |
| 29852A→T | 2 | 0 | 0 | 0 | 0 |
| 29853G→T | 1 | 0 | 0 | 1 | 0 |
| 29855T→A | 1 | 0 | 0 | 0 | 0 |
| 29856T→C | 1 | 0 | 0 | 0 | 0 |
| 29857C→T | 3 | 0 | 0 | 3 | 0 |
| 29858T→A | 0 | 0 | 248 | 0 | 0 |
| 29858T→C | 1 | 0 | 0 | 0 | 0 |
| 29858T→G | 0 | 0 | 0 | 1 | 0 |
| 29859T→A | 2 | 0 | 0 | 0 | 0 |
| 29859T→C | 0 | 0 | 0 | 8 | 0 |
| 29860A→C | 3 | 0 | 1 | 1 | 0 |
| 29860A→T | 28 | 0 | 0 | 0 | 0 |
| 29861G→A | 1 | 0 | 0 | 5 | 0 |
| 29861G→C | 0 | 0 | 1 | 0 | 0 |
| 29861G→T | 2 | 0 | 0 | 0 | 0 |
| 29862G→A | 5 | 0 | 0 | 0 | 0 |
| 29862G→C | 1 | 0 | 2 | 1 | 0 |
| 29862G→T | 1 | 0 | 0 | 17 | 0 |
| 29863A→C | 2 | 0 | 0 | 0 | 0 |
| 29863A→G | 1 | 0 | 0 | 0 | 0 |
| 29863A→T | 0 | 0 | 0 | 1 | 0 |
| 29864G→A | 5 | 0 | 1 | 0 | 0 |
| 29864G→T | 2 | 0 | 0 | 0 | 0 |
| 29865A→G | 1 | 0 | 1 | 0 | 0 |
| 29865A→T | 3 | 0 | 0 | 0 | 0 |
| 29866A→C | 1 | 0 | 0 | 0 | 0 |
| 29866A→G | 0 | 0 | 0 | 1 | 0 |
| 29867T→A | 5 | 0 | 0 | 0 | 0 |
| 29867T→C | 1 | 0 | 0 | 1 | 0 |
| 29867T→G | 4 | 0 | 0 | 0 | 0 |
| 29868G→A | 8 | 0 | 1 | 0 | 0 |
| 29868G→T | 1 | 0 | 1 | 0 | 0 |
| 29869A→C | 2 | 0 | 0 | 0 | 0 |
| 29869A→G | 1 | 0 | 1 | 0 | 0 |
| 29869A→T | 1 | 0 | 0 | 0 | 0 |
| 29870C→A | 309 | 0 | 27 | 76 | 0 |
| 29871A→C | 2 | 0 | 1 | 0 | 0 |
| 29871A→G | 4 | 0 | 0 | 1 | 0 |
| 29871A→T | 1 | 0 | 0 | 0 | 0 |
| 29872A→G | 3 | 0 | 0 | 0 | 0 |
| 29872A→T | 0 | 0 | 0 | 1 | 0 |
| 29873A→C | 1 | 0 | 0 | 0 | 0 |
| 29874A→C | 0 | 0 | 0 | 1 | 0 |
| 29875A→G | 3 | 0 | 0 | 0 | 0 |
| 29876A→C | 0 | 0 | 0 | 1 | 0 |
| 29877A→G | 2 | 0 | 0 | 0 | 0 |
| 29878A→C | 2 | 0 | 0 | 0 | 0 |
| 29880A→C | 1 | 0 | 0 | 0 | 0 |
| 29882A→C | 2 | 0 | 0 | 0 | 0 |
| 29886A→G | 1 | 0 | 0 | 0 | 0 |
| 29887A→G | 1 | 0 | 0 | 0 | 0 |
| 29896A→G | 1 | 0 | 0 | 0 | 0 |
